# Cytoprotective and Cytotoxic Effects of Rice Bran Extracts in Rat H9c2(2-1) Cardiomyocytes

**DOI:** 10.1155/2016/6943053

**Published:** 2016-04-27

**Authors:** Xian Wen Tan, Mrinal Bhave, Alan Yean Yip Fong, Eiji Matsuura, Kazuko Kobayashi, Lian Hua Shen, Siaw San Hwang

**Affiliations:** ^1^Faculty of Engineering, Computing and Science, Swinburne University of Technology Sarawak Campus, Jalan Simpang Tiga, 93350 Kuching, Sarawak, Malaysia; ^2^Swinburne Sarawak Research Centre for Sustainable Technologies, Swinburne University of Technology Sarawak Campus, Jalan Simpang Tiga, 93350 Kuching, Sarawak, Malaysia; ^3^Faculty of Science, Engineering and Technology, Swinburne University of Technology, Hawthorn, Melbourne, VIC 3122, Australia; ^4^Sarawak General Hospital Heart Centre, 94300 Kota Samarahan, Sarawak, Malaysia; ^5^Clinical Research Centre, Sarawak General Hospital, Jalan Hospital, 93586 Kuching, Sarawak, Malaysia; ^6^Collaborative Research Center (OMIC), Okayama University Graduate School of Medicine, Dentistry, and Pharmaceutical Sciences, Okayama 700-8558, Japan; ^7^Department of Cell Chemistry, Okayama University Graduate School of Medicine, Dentistry, and Pharmaceutical Sciences, Okayama 700-8558, Japan

## Abstract

This study was aimed at preliminarily assessing the cytoprotective and antioxidative effects of rice bran extracts (RBEs) from a Sarawak local rice variety (local name: “BJLN”) and a commercial rice variety, “MR219,” on oxidative stress in rat H9c2(2-1) cardiomyocytes. The cardiomyocytes were incubated with different concentrations of RBE and hydrogen peroxide (H_2_O_2_), respectively, to identify their respective IC_50_ values and safe dose ranges. Two nonlethal and close-to-IC_50_ doses of RBE were selected to evaluate their respective effects on H_2_O_2_ induced oxidative stress in cardiomyocytes. Both RBEs showed dose-dependent cytotoxicity effects on cardiomyocytes. H_2_O_2_ induction of cardiomyocytes pretreated with RBE further revealed the dose-dependent cytoprotective and antioxidative effects of RBE via an increase in IC_50_ values of H_2_O_2_. Preliminary analyses of induction effects of RBE and H_2_O_2_ on cellular antioxidant enzyme, catalase (CAT), also revealed their potential in regulating these activities and expression profile of related gene on oxidative stress in cardiomyocytes. Pretreated cardiomyocytes significantly upregulated the enzymatic activity and expression level of CAT under the exposure of H_2_O_2_ induced oxidative stress. This preliminary study has demonstrated the potential antioxidant effects of RBE in alleviating H_2_O_2_-mediated oxidative injuries via upregulation in enzymatic activities and expression levels of CAT.

## 1. Introduction

Research on plant-derived natural antioxidants has become one of the emerging fields of study in recent years [1]. Phytochemicals are natural antioxidants, comprised of phenolic or polyphenolic compounds such as polyphenols, flavonoids, anthocyanins, vitamins, and/or resveratrol, which are commonly found in fruits, vegetables, and nuts [2]. It has been demonstrated that frequent dietary intake of antioxidant-rich food is commonly linked with low incidence of oxidative stress associated diseases. These naturally occurring bioactive constituents provide a defense system to the body by eliminating free radicals and protecting the body against oxidative injury [3]. Studies on natural antioxidants have shown positive health effects towards cardioprotection, anti-inflammation, anti-infection, liver protection, antidiabetic, antiobesity, and antineurodegenerative processes [4–9]. These health benefits are proposed to be attributed to the synergistic antioxidant protective effects of different phytochemicals [10].

Natural antioxidants have been proven to quench free radicals effectively, improve the antioxidant status of cells, and provide protection against cellular oxidative injuries [11]. Interactions between oxidants and antioxidants control various crucial cellular pathways and metabolism; the simple “oxidant-antioxidant imbalance” theory has now grown to be incorporated into the progression of various chronic diseases. Hence, the rationale for strategies utilizing exogenous natural antioxidants as therapeutic intervention to attenuate cardiac injury through inhibition of inadvertent cellular oxidative damage or signaling pathways may have important implications for both the prevention and treatment of these diseases [2]. 

Rice is a staple food and remains the utmost important agricultural commodity in many Asian countries [12]. It provides the main source of calories and nourishment for the majority of the Asian population's nutritional requirement [13]. In addition, rice continues to play a significant role in sustaining global food security systems and establishing a continual capacity to feed the increasing world population [14]. The whole rice grain is known for containing rich contents of vitamins, lipids, minerals, proteins, fibres, and numerous antioxidants [15] which may aid in disease control [16]. Major composition of these bioactive compounds is found in the bran of rice grain. Several research works involving animal models have been focusing on the health attributes of rice bran in the prevention and treatment of chronic diseases. The outcomes from these studies revealed positive correlation between the consumption of rice bran and risk reductions in chronic diseases such as cardiovascular disease [17–19], cancers [20, 21], type 2 diabetes [22], hypertension, and hyperlipidaemia [23]. Through the emerging knowledge of rice bran in health and wellness, its consumption has begun to gain popularity in recent years [24]. The current research trend in rice bran revolves around its innovation in the food system that aims to alleviate issues of malnutrition and chronic diseases. In addition, emphasis is also put on the genetic, geographic, and nutritional diversities of different rice varieties and their associated health attributes [25]. By addressing all these research statements, it will provide global health prospects for proper and innovative utilization of rice bran in the management of chronic diseases. Hence in the present study, we determined the cytoprotection and the antioxidant properties of rice bran extract derived from a Sarawak local rice variety (“BJLN”) and a commercial rice variety (“MR219”).

## 2. Materials and Methods

### 2.1. Chemicals and Materials

Analytical grade methanol (MetOH) (EMSURE®) was purchased from Merck (Darmstadt, Germany). Absolute ethanol (EtOH) was purchased from Fisher Scientific (Malaysia). H9c2(2-1) cardiomyocytes of* Rattus norvegicus* rat (ATCC® CRL-1446*™*) were purchased from ATCC. CellTiter 96® Aqueous Non-Radioactive Cell Proliferation Assay Kit was purchased from Promega; Dulbecco's Modified Eagle Medium (DMEM) and phosphate buffer saline (PBS) were purchased from Gibco®. Fetal Bovine Serum (FBS), penicillin-streptomycin (10,000 units), 0.25% trypsin-EDTA, trypan blue, and dimethyl sulfoxide (DMSO) were purchased from Sigma Aldrich. AxyPrep Multisource Total RNA Miniprep Kit was purchased from Axygen. QuantiFast SYBR® Green RT-PCR Kit was purchased from Qiagen. Catalase Assay Kit was purchased from Cayman Chemical.

### 2.2. Methodology

#### 2.2.1. Preparation of Rice Bran Extracts (RBEs)

Preparation of RBE was carried out at a sample-mass-to-solvent ratio of 1 : 10 (gram to millilitres), using 3 g of rice bran and 30 mL of analytical grade methanol. The mixture was stirred continuously on a stirring hot plate (Stirring Hot Plate HS0707V2, Favorit) for 30 minutes, at room temperature. After 30 minutes, the RBEs were centrifuged (Centrifuge 5702, Eppendorf) for 10 minutes at 1,000 RPM. The supernatants were collected and extraction of the residual bran samples was repeated twice more and all the supernatants were combined.

The solvents in the collected extracts were then evaporated using rotary evaporator at 35°C (RE300, Yamato) and further concentrated using vacuum concentrator (7810037, Labconco) until they were fully lyophilized. The lyophilized extracts were then weighed and kept in a −22°C freezer until further use. The lyophilized extract samples were dispersed in absolute ethanol to prepare crude extracts, each with a known mass concentration. These prepared stocks were then used to prepare a series of diluted (2x dilution) samples.

### 2.3. Cell Culture

H9c2(2-1) cardiomyocytes of* Rattus norvegicus* rat were used as the mammalian cell culture model for the antioxidant assay. The cells were cultivated in DMEM media supplemented with 10% FBS and 100 units/mL of penicillin-streptomycin (final concentration). Cells were incubated at 37°C and 5% CO_2_. Subcultivation of cells was performed when cells achieved 70%–80% confluency. Cells in passages numbers 20–25 were used in all experiments and cells (7.5 × 10^3^ cells/per well) were seeded on a 96-well microplate for different experiments.

### 2.4. Cell Cytotoxicity Assay

Cell toxicity effects of selected RBE and H_2_O_2_ were studied by using a 3-(4,5-dimethylthiazol-2-yl)-5-(3-carboxymethoxyphenyl)-2-(4-sulfophenyl)-2H tetrazolium- (MTS-) based assay kit (CellTiter 96 Aqueous Non-Radioactive Cell Proliferation Assay Kit, Promega). Approximately 7.5 × 10^3^ cells were plated onto each well of a 96-well microplate and preincubated for 24 hours before the cells were further treated with RBE.

#### 2.4.1. Cell Cytotoxicity Study of RBE

Different concentrations (approximately 6.25 *μ*g/mL to 500 *μ*g/mL) of selected RBE were prepared by serially diluting the prepared stocks in twofold dilutions with serum-free DMEM. The final concentration of ethanol content in each sample was kept below 1% (v/v), and media containing ethanol (1% v/v) were used as negative control in the assays. Three separate sets of experiments were set up to study the time-dependent cytotoxicity effects of RBE treated cells over the duration of 24, 48, and 72 hours, respectively. Cell viability was determined via the MTS assay kit (CellTiter 96 Aqueous Non-Radioactive Cell Proliferation Assay Kit, Promega). Briefly, after the incubation period, cells were washed with PBS buffer and later replenished with fresh serum-free DMEM. MTS reagent was then added to each well and the microplate was incubated for 4 hours before the absorbance was measured at 490 nm through a microplate reader (Synergy HT, Biotek). Cell cytotoxicity effects of rice bran extracts were determined by identifying the dosage decreasing the cell viability by 50% of the initial population.

#### 2.4.2. Cell Cytotoxicity Study of Hydrogen Peroxide (H_2_O_2_)

Different concentrations (approximately 15 *μ*M to 1000 *μ*M) of H_2_O_2_ were prepared by serially diluting the prepared stock (1000 *μ*M) in twofold dilutions with PBS buffer. The range of concentrations was prepared to identify the dose-dependent cell cytotoxicity effects of H_2_O_2_. Standardisation of H_2_O_2_ was performed spectrophotometrically by measuring the absorbance of prepared samples at 240 nm, and a molar extinction coefficient of 43.6 M^−1 ^cm^−1^ was used to calculate the actual concentration of H_2_O_2_ prepared. PBS buffer was used as negative control in the assay. H_2_O_2_ treated cells were incubated for 24 hours and cell viability was determined via the MTS assay kit (CellTiter 96 Aqueous Non-Radioactive Cell Proliferation Assay Kit, Promega). Briefly, after the incubation period, media were discarded and cells were washed and replaced with fresh serum-free DMEM. MTS reagent was then added to each well and the microplate was incubated for 4 hours before the absorbance was measured as above.

### 2.5. Cytoprotective Effects of RBE on Oxidative Stress Induced Cells

H9c2(2-1) cells were seeded and incubated at 37°C and 5% CO_2_ for 24 hours before they were treated with RBE. The cells were treated with specific concentrations of the selected RBE and were incubated for 24 hours. After 24 hours of incubation, growth media were replaced and oxidative stress was induced by treating the cells with different concentrations (approximately 62.5 *μ*M to 1000 *μ*M) of H_2_O_2_. The treated cells were incubated for another 24 hours before the cell viability was determined via MTS assay kit (CellTiter 96 Aqueous Non-Radioactive Cell Proliferation Assay Kit, Promega). MTS reagent was then added to each well and the microplate was incubated for 4 hours before the absorbance was measured at 490 nm as above.

### 2.6. Endogenous Antioxidant Enzyme Activity Study

Catalase (CAT) was the targeted endogenous antioxidant enzyme in this experiment. The activity of CAT was studied by using commercially available ELISA kit. Samples were prepared as per protocols stated in the kits' manual. Briefly, cells were detached by using rubber policeman and collected in ice cold PBS buffer (pH 7.4). Cell lysis was performed via physical disruption by sonicating the cells in ultrasonic water bath for 2 minutes. The CAT activity was examined via Catalase Assay Kit (Cayman Chemical). The absorbances of reaction mixtures were measured at respective wavelength defined for the assay kit.

### 2.7. Endogenous Antioxidant Enzyme Gene Expression Study

Catalase (CAT) (catalase, Cat (Gene ID: 24248)) was the targeted endogenous antioxidant enzyme in this experiment. The effects of RBE and H_2_O_2_ inductions on the gene expression of targeted endogenous antioxidant enzyme, CAT, were assessed through quantitative Real Time Polymerase Chain Reaction (qRT-PCR) approach. H9c2(2-1) cells were seeded and incubated at 37°C and 5% CO_2_ on 6-well plates for 24 hours before they were treated with RBE and H_2_O_2_, respectively.

#### 2.7.1. Total RNA Extraction

Extraction of RNA from H9c2(2-1) cardiomyocytes was performed through AxyPrep Multisource Total RNA Miniprep Kit (Axygen Biosciences). Prior to RNA extraction, supernatants were discarded and cells were washed twice with ice cold PBS buffer (pH 7.4). Then, the extraction of RNA from cells was performed as described in the kit protocol. RNase-free water was used to elute the purified total RNA. RNA samples were kept on ice when in use or stored at −80°C until further use.

#### 2.7.2. Nucleic Acid Quantitation and Qualification

The concentration and purity of extracted RNA were assessed spectrophotometrically through a microplate reader (Synergy HT, Biotek) by using Take 3 Micro-Volume Plates. The preset settings for nucleic acid quantitation and qualification were selected, and the absorbances of samples were measured at the wavelengths of 230 nm, 260 nm, and 280 nm, with a background check at 320 nm (background check). RNase-free water was used as blank reagent. The absorbance ratios of 260/280 and 260/230 were used to determine the purity of RNA samples. The acceptable absorbance ratio for 260/280 as pure RNA is ≥2.0 while that for 260/230 is 2.0 to 2.2 ref.

#### 2.7.3. Relative Quantitation of Gene Expression

Gene expression studies of targeted endogenous cellular antioxidant enzymes were performed through qRT-PCR approach. A one-step qRT-PCR kit (QuantiFast SYBR Green RT-PCR Kit, Qiagen) was used to quantify the RNA targets. A total of 20 ng of RNA sample (final amount per reaction tube = 2 ng) was mixed with reagent kits and oligonucleotide primer sets as per manufacturer's instructions. The primers used in this experiment were as follows:

 
*Catalase (CAT)*
 (Gene ID: 24248)
 Species:* Rattus norvegicus*
   
*Forward Primer*:
   5′-CGCCTGTGTGAGAACATTGC-3′
   
*Reverse Primer*:
   5′-TAGTCAGGGTGGACGTCAGT-3′
 
*Glyceraldehyde 3-phosphate dehydrogenase (GAPDH)*
 (Gene ID: 24383)
 Species:* Rattus norvegicus*
   
*Forward Primer*:
   5′-CAG GGC TGC CTT CTC TTG TG-3′
   
*Reverse Primer*:
   5′-CTT GCC GTG GGT AGA GTC AT-3′

Amplification reactions of RNA targets were performed via Rotor-Gene Q 2plex HRM Platform (Qiagen). Settings for reaction cycles were configured as specified in the kit manual. All reactions were normalized to mRNA expression of the housekeeping gene, GAPDH for* Rattus norvegicus*. All samples were prepared in triplicate and relative gene expression levels of RNA targets were normalized to that of negative control cells.

### 2.8. Statistical Analysis

All results data are presented as mean and standard deviation of three consecutive technical repetitions on the statistical tool; GraphPad Prism (GraphPad Software, Inc., USA) was used to analyse the data via one-way analysis of variance (ANOVA) and Student's *t*-test. Statistical significance and confidence level of data are set at *P* ≤ 0.05.

## 3. Results and Discussion

### 3.1. Assessment of Cell Cytotoxicity Effects of Rice Bran Extracts (RBEs)

**Figure 1:**
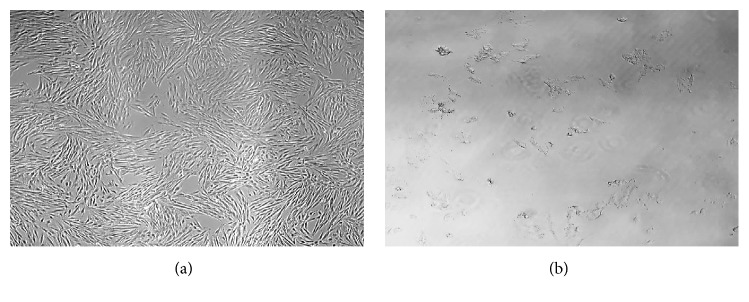
Changes in cellular morphology of H9c2(2-1) cardiomyocytes resulting from treatment with RBE. (a) Untreated cells (negative control, with 1% EtOH); (b) cells treated with BJLN RBE (500 *μ*g/mL) (Nikon Eclipse T*i*-S; mag 40x).

Figure 1 shows the microscopy images (magnification: 40x) of untreated (negative control) and treated H9c2(2-1) cells. The H9c2(2-1) cells were treated with 500 *μ*g/mL RBE of BJLN in the cell culture medium, while the negative control cells were treated with 1% (v/v) EtOH. The 1% (v/v) EtOH did not induce any cytotoxic effect (Figure 1(a)) on the cells. The cells were found to be thin and elongated and appear to be multinucleated (having multiple nuclei in a cell), as expected for muscle cells. In contrast, H9c2(2-1) cells treated with 500 *μ*g/mL RBE of BJLN induced distorted cell morphologies, with disintegration of cell membrane and nuclei (Figure 1(b)), and cellular debris spreading across the surface of the cell culture flask. The observations indicate that the RBE of BJLN is cytotoxic to H9c2(2-1) cells at dosage of 500 *μ*g/mL with cell viability significantly dropped to only 18.58% (*P* < 0.01) as compared to negative control.

**Figure 2:**
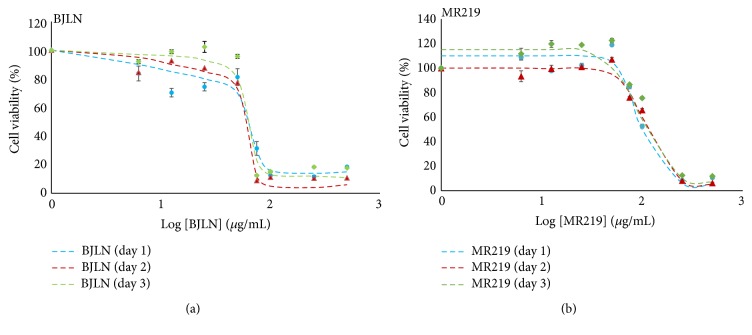
Cell viability of H9c2(2-1) cardiomyocytes treated with RBE. The H9c2(2-1) cardiomyocytes were treated with different concentrations (approximately 6.25 *μ*g/mL to 500 *μ*g/mL) of BJLN and MR219 RBE over 24 (blue circle), 48 (red triangle), and 72 (green diamond) hours of incubation time, respectively. Best fit curves drawn by using Excel.

H9c2(2-1) cardiomyocytes were induced with different concentrations of RBE (6.25 *μ*g/mL–1000 *μ*g/mL) over 24, 48, and 72 hours of incubation to identify their respective safe dose range. Cell toxicities of selected RBE were examined via MTS-based assay kit, which measures the metabolic rate of mitochondrial activities through conversion of MTS to formazan by viable cells [26]. Data are presented in terms of relative cell viability versus log of extract dosage (Figure 2).

Dose-dependent cytotoxicity was observed in cells treated with RBE of BJLN and MR219. For RBE of BJLN, extract concentrations beyond 75 *μ*g/mL induced cell death, with cell viabilities dropping below 32% (Table 1). Cell viabilities dropped below 20% after 48 and 72 hours. However, viabilities of H9c2(2-1) cells treated with BJLN RBE in the range of approximately 6.25 *μ*g/mL to 50 *μ*g/mL were >70% throughout the three incubation times. In accordance with the International Organization for Standardization (ISO), ISO 10993-5, cellular response with cell viability that falls within 70% and above is considered noncytotoxic [27]. With regard to the obtained result, it indicated that the safe working concentration range of this extract is up to 50 *μ*g/mL. The half maximal inhibitory concentration (IC_50_) of RBE refers to the concentration of RBE required for the inhibition of cell viability by 50% in comparison to negative control cells [28]. Based on the results (Table 2), the IC_50_ values of RBE of BJLN were in the range of 61.67 to 64.57 *μ*g/mL over 24, 48, and 72 hours of incubation time.

For RBE of MR219, concentrations >250 *μ*g/mL induced critical cell death, with H9c2(2-1) cell viabilities dropping below 13% (Table 1). Cell viabilities further dropped below 12% with 500 *μ*g/mL after 24, 48, and 72 hours of incubation. Contrarily, viabilities of cells treated with MR219 extract in the range of 6.25 to 75 *μ*g/mL were >70% throughout the three different incubation periods. Therefore, the safe concentration range of MR219 extract appears to be 6.25–75 *μ*g/mL (Table 1). Based on the results, the IC_50_ values of MR219 RBE were in the range of 95.44 to 111.50 *μ*g/mL over the three different incubation periods (Table 2).

The results showed the dose-dependent cytotoxic effects of BJLN and MR219 extracts on H9c2(2-1) cardiomyocytes. In general, cell viabilities dropped below 20% when high doses (>250 *μ*g/mL) of RBE were used. Induction of cells with RBE within the range of safe dosage showed improvements in cell viabilities with longer incubation period. The possible reasons for such observations could be the potential cell proliferation induction effects from the extracts or activation of cellular protective response that counteract the stress, for adaptation and survival. However, further studies are needed to support this conjecture. The data also revealed that the IC_50_ range of RBE of BJLN was lower than that for MR219.

**Figure 3:**
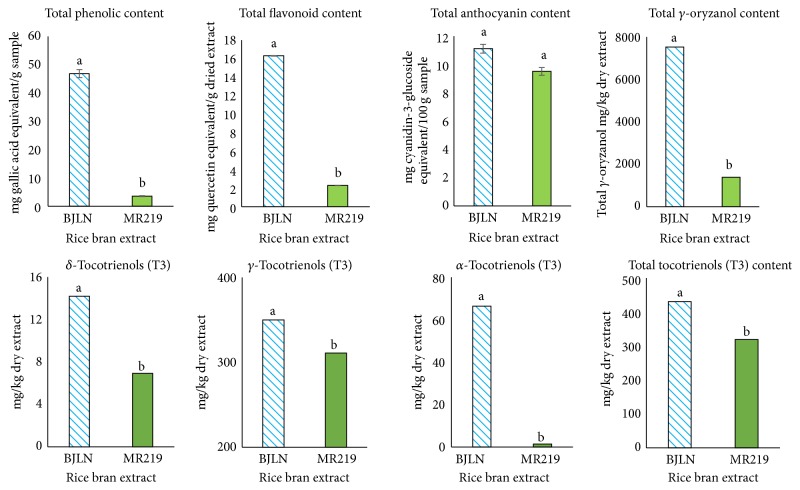
Total contents of selected bioactive compounds in the RBE. Different letter on a bar represents significant differences at *P* ≤ 0.05 (Tukey's test).

Chemical analyses of total antioxidant compound contents indicated that the RBE of BJLN had significantly higher contents of antioxidants (total phenolic and total flavonoid compounds, total *γ*-oryzanol, and total vitamin E components) compared to MR219 (Figure 3). Therefore, the RBE of BJLN may require a lower concentration to achieve similar antioxidant activities as the MR219. These differences may account for the difference in their IC_50_.

Significant decrement in cell viability of H9c2(2-1) cardiomyocytes was reported when high doses of RBE were used, suggesting dose-dependent cytotoxicity effects of the RBEs. Several reports have highlighted cell cytotoxicity effects of polyphenols when high doses of the antioxidants were used [29, 30] in which they act as prooxidants that threaten survival and viability of cells.

### 3.2. Assessment of Cell Cytotoxicity Effects of Hydrogen Peroxide

**Figure 4:**
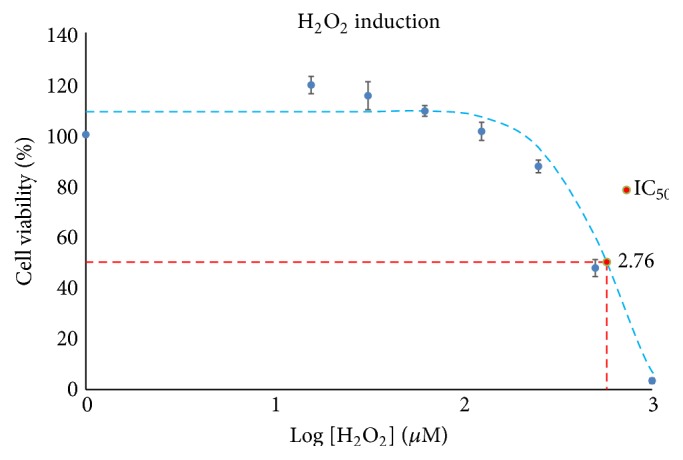
Cell viability curves of H9c2(2-1) cells treated with different concentrations of hydrogen peroxide (H_2_O_2_). The insets showed the inhibition concentration (IC_50_) of H_2_O_2_ on H9c2(2-1) cells determined via GraphPad Prism (GraphPad Software, Inc., USA). Best fit curve was drawn using Excel for visual purpose. Tabulated data are presented in Table A, in Supplementary Material available online at http://dx.doi.org/10.1155/2016/6943053.

H9c2(2-1) cardiomyocytes were induced with different concentrations of hydrogen peroxide (H_2_O_2_) to identify the suitable range of working concentrations that do not induce cell death.  Figure 4 shows the cell viability curve of H9c2(2-1) cells treated with different concentrations of H_2_O_2_. Data are presented in terms of relative cell viability versus log of extract dosage. A dose-dependent cytotoxicity effect was observed in cells treated with H_2_O_2_. The range of H_2_O_2_ concentrations between 15.63 *μ*M and 250 *μ*M did not decrease the viability of H9c2(2-1) cells. Therefore, H_2_O_2_ in this range was considered safe.

Cell viabilities were more than 87% after treatment with H_2_O_2_ within the above range. However, exceeding the concentration of 250 *μ*M H_2_O_2_ caused a significant decrease in cell viability to less than 48%. In addition, IC_50_ of H_2_O_2_ on H9c2(2-1) cells was detected at 572.10 *μ*M (log [H_2_O_2_] = 2.76).

Under normal cellular metabolic activities, low concentrations of H_2_O_2_ are produced as a by-product that is relatively harmless and beneficial to most cells [31]. Cells utilize H_2_O_2_ for processes such as oxidative biosynthesis and host defense. In addition, there are also evidences showing the potential of H_2_O_2_ as a signaling messenger in cellular signal transduction pathways [31]. However, overaccumulation of H_2_O_2_ can be deleterious, as it can lead to the onset of oxidative stress and subsequently oxidative stress mediated diseases over time [32].

Based on the results, H9c2(2-1) cells induced with low concentrations of H_2_O_2_ (15.63 *μ*M and 31.25 *μ*M) showed proliferative effect. The cell viabilities were more than 100% in relation to the negative control. This observation suggests the potential of low concentration of H_2_O_2_ in stimulating cell growth of H9c2(2-1) as low concentration of H_2_O_2_ has been reported to be capable of stimulating cell proliferation [33]. In addition, the present data are in agreement with the general response trend of proliferating mammalian cells to H_2_O_2_ [34–36]. It has been reported that low concentration of H_2_O_2_ in range of 3 to 15 *μ*M has the potential of inducing growth stimulation while the higher concentration range, 120 to 150 *μ*M, can cause growth arrest temporarily. Growth arrest may occur permanently when cells are induced with H_2_O_2_ in the concentration range between 250 *μ*M and 400 *μ*M while concentration of H_2_O_2_ beyond 1000 *μ*M may induce cell necrosis [34–36]. Therefore, variation in cellular responses towards different concentrations of H_2_O_2_ could have potentially induced the dose-dependent cytotoxicity effect of H_2_O_2_ on H9c2(2-1) cells.

### 3.3. Cytoprotective Effects of RBE on Oxidative Stress Induced Cells

**Figure 5:**
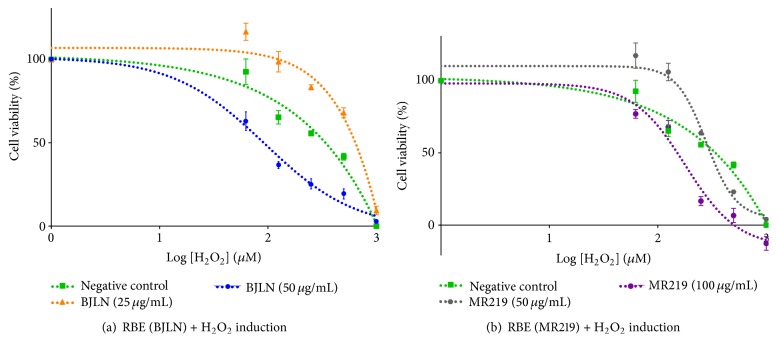
Effects of H_2_O_2_ inductions on cell viabilities of H9c2(2-1) cardiomyocytes pretreated with different concentrations of (a) BJLN RBE (25 *μ*g/mL and 50 *μ*g/mL) and (b) MR219 RBE (50 *μ*g/mL and 100 *μ*g/mL).

The potential of RBE to alleviate oxidative stress in H9c2(2-1) cells mediated by H_2_O_2_ was investigated by pretreating the cells with different concentrations of each BJLN (25 *μ*g/mL and 50 *μ*g/mL) and MR219 (50 *μ*g/mL and 100 *μ*g/mL) extract before subsequent induction with various concentrations of H_2_O_2_. The induction effects are shown in Figure 5 and Table 3. Dose-dependent cytoprotective effects against H_2_O_2_ induced cell cytotoxicity were observed in cells pretreated with RBE. The positive effects were more distinctive with lower concentrations of RBE (BJLN: 25 *μ*g/mL; MR219: 50 *μ*g/mL) with observable increments in IC_50_ of H_2_O_2_ (BJLN: 645.65 *μ*M; MR219: 320.63 *μ*M) (Table 4) when compared to negative control (316.23 *μ*M). When the two extracts were compared, BJLN (25 *μ*g/mL) extract outran MR219 (50 *μ*g/mL) extract in terms of efficacy with a significant increment in IC_50_ of H_2_O_2_ approximately twofold (645.65 *μ*M) versus 1.4% (in approximation) when compared to negative control (316.23 *μ*M). The differences in cytoprotective efficacies of both extracts could be attributed to the difference in their respective total antioxidant contents.

However, the higher concentrations of BJLN (50 *μ*g/mL) and MR219 (100 *μ*g/mL) extracts tested did not result in cellular cytoprotection towards H_2_O_2_ induced cell cytotoxicity. Significant decrements in IC_50_ values of H_2_O_2_ were found for cell pretreated with 50 *μ*g/mL BJLN (92.90 *μ*M) and 100 *μ*g/mL MR219 (171.79 *μ*M) extracts when compared to negative control (316.23 *μ*M) (Table 4). The higher concentrations of BJLN and MR219 extracts selected were near the range of IC_50_ of both extracts (IC_50_ of BJLN: 52.18 *μ*g/mL to 73.09 *μ*g/mL; IC_50_ of MR219: 95.44 *μ*g/mL to 111.50 *μ*g/mL). It was deduced that H9c2(2-1) cells could have experienced cytotoxic stress from both high concentrations of RBE and H_2_O_2_, respectively. As overdoses of natural antioxidants have been reported to exhibit prooxidant-like characteristics that potentially threaten cell survival and viability [37–39], additional cytotoxic stress derived from H_2_O_2_ could have further decreased the viability of H9c2(2-1) after treatment with extract and H_2_O_2_, respectively.

Various chronic diseases such as cardiovascular diseases, cancer, and diabetes are closely associated with oxidative stress. Factors such as molecular targets, mechanism, and severity of oxidative stress define the consequence of oxidative stress injury on cells. This may further initiate signal transduction cascade reactions that lead to the onset and progression of chronic diseases [11, 40]. The present preliminary results have revealed the potential of RBE as a source of natural antioxidants to alleviate oxidative stress mediated cytotoxicity. Coupled with further carefully planned investigations, RBE could be considered for further application as nutraceuticals for protection against chronic diseases mediated by oxidative stress, such as cardiovascular diseases.

### 3.4. Determination of the Effects of Different Cellular Inductions on Activities of Cellular Endogenous Antioxidant Enzymes and Expression of Relevant Genes

The antioxidative protective mechanism of RBE was initially indistinct at the commencement of this study. It was hypothesized that RBE could exert its antioxidative properties through induction of endogenous cellular antioxidant enzymes. Endogenous cellular enzymatic antioxidants often refer to cellular antioxidant enzymes such as superoxide dismutase (SOD), catalase (CAT), and glutathione peroxidase (GPx) [41]. They are the first line of enzyme-based cellular defensive systems and each enzyme plays a different role in alleviation of oxidative injury [42].

As CAT is known as the main regulator for catalytic decomposition of H_2_O_2_ to harmless water molecules (H_2_O) and oxygen (O_2_) [43], it was selected as the biomarker to test the above-mentioned hypothesis. The antioxidative protective effect of RBE was tested against the CAT enzyme, of which the effects of different inductions were assessed by studying changes in expression of the corresponding gene and total cellular activities of CAT on H9c2(2-1) cells subjected to different treatments as compared to those in untreated (control) cells.

#### 3.4.1. Effects of RBE Inductions on Activities and Expression of CAT Gene

**Figure 6:**
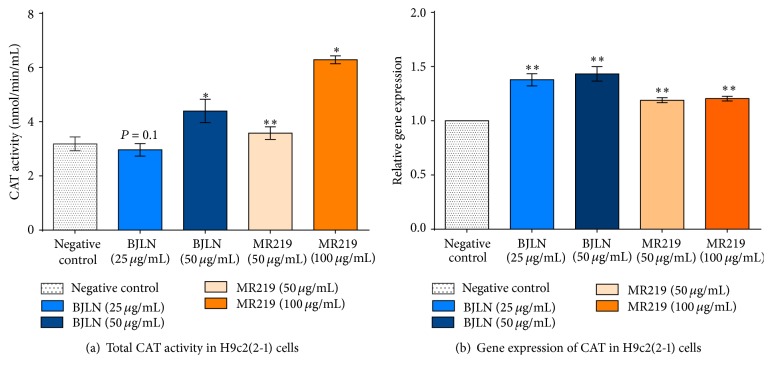
Effects of RBE pretreatment of cells on CAT enzyme and gene expression. (a) Total CAT enzymatic activity; (b) CAT (catalase, Cat (Gene ID: 24248)) gene expression levels in H9c2(2-1) cells pretreated with different concentrations of RBE. Data represent mean ± standard deviation of three technical replicates (*n* = 3).  ^*∗*^Significantly different from negative control (*P* ≤ 0.05);  ^*∗∗*^significantly different from negative control (*P* ≤ 0.01).

Two extract concentrations that did not induce cell cytotoxicity were selected to study their respective induction effects on gene activities and gene expression of CAT in induced H9c2(2-1) cardiomyocytes. The effects of treating H9c2(2-1) cells with RBE on the enzymatic activity and expression level of CAT are depicted in Figure 6. Induction with BJLN (50 *μ*g/mL) and MR219 (50 and 100 *μ*g/mL) RBE appeared to have elevated the enzymatic activity of CAT, while no significant improvement was observed with 25 *μ*g/mL of BJLN (Figure 6(a)). Higher concentrations of RBEs typically induced higher activities. In addition, significantly higher activities were observed with MR219 RBE compared to BJLN. Briefly, the activity had increased by ~40% and ~100% in relation to negative control with 50 *μ*g/mL of BJLN RBE and 100 *μ*g/mL of MR219 RBE, while 50 *μ*g/mL of MR219 RBE only weakly elevated the activity (~16%). The CAT (catalase, Cat (Gene ID: 24248)) gene expression levels were also significantly upregulated with all concentrations of extracts (Figure 6(b)) in the range of ~18% to ~40%. In addition, it was discovered that BJLN extracts expressed higher levels of CAT (relative to negative control) as compared to MR219 extracts. There was no significant difference in expression of CAT between the two different concentrations of each extract selected for this part of the study. The effects of RBEs on the enzymatic activity and gene expression of CAT may be attributed to their polyphenol contents, as these can generate prooxidants. This may cause oxidative stress and trigger cytoprotective mechanisms.

#### 3.4.2. Effects of H_2_O_2_ Inductions on Activities and Expression of CAT

**Figure 7:**
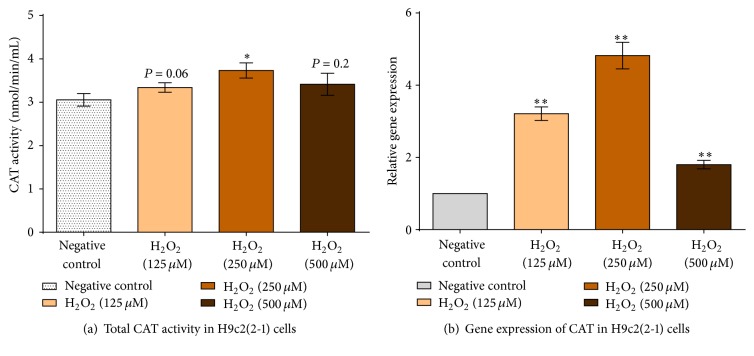
(a) Total enzymatic activities and (b) gene expression levels of CAT after induction with different concentrations of H_2_O_2_. Data represent mean ± standard deviation of three technical repetitions (*n* = 3). “*∗*”: significantly different from negative control at *P* ≤ 0.05; “*∗∗*”: significantly different from negative control at *P* ≤ 0.01.

Effects of H_2_O_2_ inductions on the enzymatic activity and expression levels of CAT in H9c2(2-1) cells were depicted in Figure 7. H9c2(2-1) cells were incubated with three different concentrations of H_2_O_2_ for 24 hours. The results revealed that cellular induction with 250 *μ*M of H_2_O_2_ significantly increased the CAT activity by ~20%. Contrarily, there no significant difference in CAT activity was observed with 125 *μ*M and 500 *μ*M of H_2_O_2_, respectively (Figure 7(a)). Induction of H9c2(2-1) with different concentrations of H_2_O_2_ significantly elevated the expression levels of CAT (Figure 7(b)), ~80% to ~380% in comparison to negative control. These observations were concurrent with other reported studies [44–47]. Briefly, an increment in expression and activity of catalase represents the cellular defense mechanism against H_2_O_2_-mediated oxidative injuries. Among the three different concentrations of H_2_O_2_ studied (125 *μ*M, 250 *μ*M, and 500 *μ*M), the highest upregulation was with 250 *μ*M H_2_O_2_ (4.8-fold), followed by 125 *μ*M H_2_O_2_ (3.2-fold) and 500 *μ*M H_2_O_2_ (1.8-fold).

CAT was actively involved in the detoxification of H_2_O_2_ produced from the enzymatic reaction of SOD and cellular metabolic activities [41]. It catalyses the conversion of H_2_O_2_ to H_2_O and O_2_ in a two-step reaction [48]. CAT naturally has a high Michaelis constant (Km) for H_2_O_2_; hence it is capable of neutralizing high concentration of H_2_O_2_ [49]. In the presence of different concentrations of exogenous H_2_O_2_, activities and gene expression of CAT in H9c2(2-1) cardiomyocytes induced with different concentrations of H_2_O_2_ were significantly upregulated in a dose-dependent manner. An increase in activity and expression levels of CAT was reported with 125 *μ*M and 250 *μ*M of H_2_O_2_, respectively, and followed by a decrease in activity and gene expression of CAT when cells were induced with 500 *μ*M H_2_O_2_.

#### 3.4.3. Effects of H_2_O_2_ Inductions on Activities and Expression of CAT in Cells Pretreated with RBE

**Figure 8:**
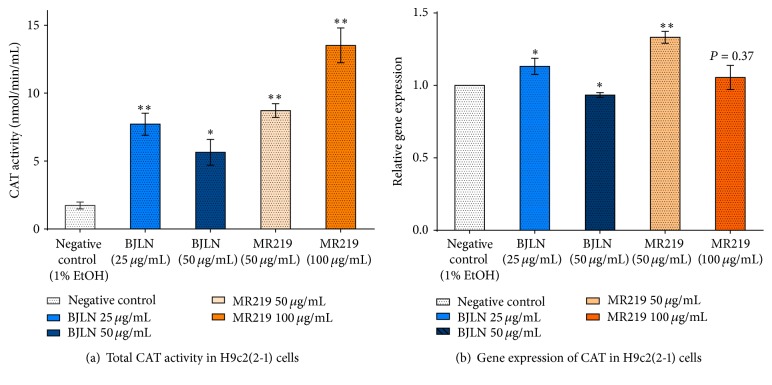
(a) Total enzymatic activities and (b) gene expression levels of CAT in RBE pretreated H9c2(2-1) cells after induction with 125 *μ*M of H_2_O_2_. Data represent mean ± standard deviation of three technical repetitions (*n* = 3). “*∗*”: significantly different from negative control (*P* ≤ 0.05); “*∗∗*”: significantly different from negative control (*P* ≤ 0.01).

Figure 8 showed the effects of the treatment on enzymatic activities and gene expression of CAT. Briefly, CAT activities of cells pretreated with RBE were significantly increased (3–8-fold) after being induced with 125 *μ*M of H_2_O_2_ (Figure 8(a)). Sample group pretreated with 100 *μ*g/mL of MR219 extract reported the highest fold change (~8-fold) in CAT activity when compared to control group, followed by 50 *μ*g/mL of MR219 extract (~5-fold), 25 *μ*g/mL of BJLN extract (~4.5-fold), and 50 *μ*g/mL of BJLN extract (~3.3-fold). With regard to the gene expression profiles of CAT, the lower concentrations of both BJLN (25 *μ*g/mL) and MR219 (50 *μ*g/mL) extracts significantly upregulated the expression levels of CAT by 13% and 33%, respectively, in relation to negative control. A slight downregulation in expression of CAT (~7%) was observed with cells pretreated with 50 *μ*g/mL of BJLN extract. No significant difference in expression of CAT was noted between negative control and 100 *μ*g/mL of MR219 treated cells. It is of note that there was a lack of exact correlation between the enzymatic activity of CAT and its gene expression level. Such findings were also reported by Chiang et al. whose work was focusing on evaluating the activities of SOD, CAT, and GPx in HepG2 cells treated with rice extracts [50]. Similarly, it was reported that there was a lack of exact relationship between the enzymes' activities and their respective gene expression [50]. It was suggested that such discrepancy was possibly regulated posttranslationally [51].

Based on the above findings, induction of H9c2(2-1) cardiomyocytes with different concentrations of RBE and H_2_O_2_ revealed their distinctive effects in regulating the activities of CAT. The present data revealed that pretreating H9c2(2-1) cells with RBE before H_2_O_2_ induction (with 125 *μ*M H_2_O_2_) resulted in significant improvement in the enzymatic activity of CAT. It is proposed that RBE could have protected H9c2(2-1) cells from oxidative injuries mediated by H_2_O_2_ via upregulation of CAT activity. However, other mechanisms involved in antioxidative properties of RBE against oxidative assault mediated by H_2_O_2_ remain to be further elucidated. Although present findings showed promising regulatory effect of RBE in the enzymatic activity of CAT, extended investigation on other additional biomarkers for intracellular ROS levels, apoptotic or necrotic cell death, or cell signaling pathways is suggested for further studies. The latter will offer deeper insight into the protective mechanism of RBE against H_2_O_2_-mediated cell cytotoxicity.

## 4. Conclusion

In the present study, the antioxidant activities of RBE derived from Sarawak local rice variety (BJLN) and a commercial rice variety (MR219) have been studied via* in vitro* cell-based assays. The results have demonstrated the potential of RBE as a source of naturally derived antioxidants to alleviate oxidative stress mediated cytotoxicity. With further investigations, RBE could be considered for application as a nutraceutical for protection against chronic diseases mediated by oxidative stress.

## Supplementary Material

Table A represents the data for cell viability of H9c2(2-1) after inductions with different concentrations of hydrogen peroxide (H_2_O_2_). Data presented were the mean ± standard deviation of three replicates (*n*=3). ‘∗' on each column denotes significant differences at *P*≤0.05 as compared to negative control. Graphical representation of data is illustrated in Fig. 4.

## Figures and Tables

**Table 1:** Cell viability of H9c2(2-1) after induction with RBE of BJLN and MR219.

Log (dose)	Dose (*µ*g/mL)	Cell viability (%)					
		BJLN			MR219		
		Day 1 (24 hours)	Day 2 (48 hours)	Day 3 (72 hours)	Day 1 (24 hours)	Day 2 (48 hours)	Day 3 (72 hours)
0.80	6.25	83.76 ± 5.18	84.47 ± 1.45^*∗*^	91.96 ± 2.70^*∗*^	108.88 ± 4.76	93.34 ± 8.97	111.70 ± 5.63
1.10	12.5	70.33 ± 5.95^*∗*^	92.55 ± 2.38^*∗*^	98.93 ± 2.75	97.78 ± 1.21	99.48 ± 5.67	119.73 ± 5.63^*∗*^
1.40	25	74.42 ± 5.79^*∗*^	87.51 ± 5.77	102.32 ± 7.60	102.22 ± 3.44	101.05 ± 1.44	118.93 ± 1.61^*∗*^
1.70	50	81.19 ± 5.86^*∗*^	77.23 ± 3.02	95.71 ± 2.75	118.93 ± 1.42^*∗*^	107.03 ± 3.41	122.50 ± 3.65^*∗*^
1.88	75	31.37 ± 4.83^*∗*^	8.81 ± 0.00^*∗*^	12.50 ± 1.12^*∗*^	84.99 ± 3.68^*∗*^	76.08 ± 1.27^*∗*^	86.61 ± 2.64^*∗*^
2.00	100	12.44 ± 0.74^*∗*^	11.23 ± 0.48^*∗*^	15.14 ± 1.29^*∗*^	52.57 ± 3.21^*∗*^	65.79 ± 2.38^*∗*^	75.54 ± 3.21^*∗*^
2.40	250	11.68 ± 1.61^*∗*^	10.49 ± 0.66^*∗*^	18.33 ± 1.09^*∗*^	8.41 ± 0.61^*∗*^	8.18 ± 0.55^*∗*^	12.50 ± 1.12^*∗*^
2.70	500	18.57 ± 0.61^*∗*^	10.70 ± 0.94^*∗*^	17.68 ± 0.00^*∗*^	10.63 ± 1.13^*∗*^	6.30 ± 1.09^*∗*^	11.79 ± 1.86^*∗*^

Data represented as mean ± standard deviation of three technical replicates (*n* = 3). ^*∗*^Significant difference at *P* ≤ 0.05 as compared to negative control.

**Table 2:** The inhibitory concentration (IC_50_) of RBE of BJLN and MR219. Data presented as mean ± standard deviation of three technical replicates (*n* = 3).

	BJLN		MR219	
	Log (dose) (*µ*g/mL)	Dose (*µ*g/mL)	Log (dose) (*µ*g/mL)	Dose (*µ*g/mL)
Day 1 (24 hours)	1.81 ± 0.01	64.57 ± 0.91	1.98 ± 0.01	95.44 ± 1.02
Day 2 (48 hours)	1.79 ± 0.002	61.67 ± 0.32	2.05 ± 0.03	111.50 ± 1.07
Day 3 (72 hours)	1.80 ± 0.04	63.10 ± 4.99	2.03 ± 0.02	107.20 ± 1.05

**Table 3:** Cell viability of H9c2(2-1) after inductions with different concentrations of H_2_O_2_. Cells were pretreated with different concentrations of BJLN and MR219 RBE before H_2_O_2_ induction. Data represent mean ± standard deviation of three technical replicates (*n* = 3). “*∗*” on each column denotes significant differences at *P* ≤ 0.05 as compared to negative control (nontreated cells).

Log [H_2_O_2_], *µ*M	H_2_O_2_ (*µ*M)	Cell viability (%)				
		Negative control (media + 1% EtOH)	BJLN (25 *µ*g/mL)	BJLN (50 *µ*g/mL)	MR219 (50 *µ*g/mL)	MR219 (100 *µ*g/mL)
1.80	62.5	92.61 ± 7.62	116.67 ± 4.83^*∗*^	62.80 ± 5.55^*∗*^	117.30 ± 8.43	76.93 ± 8.30^*∗*^
2.10	125	65.31 ± 3.75^*∗*^	99.09 ± 6.12	36.95 ± 2.09^*∗*^	105.91 ± 6.24	67.93 ± 2.96^*∗*^
2.40	250	55.70 ± 1.27^*∗*^	83.05 ± 1.61^*∗*^	25.04 ± 3.22^*∗*^	63.50 ± 0.97^*∗*^	16.86 ± 2.88^*∗*^
2.70	500	41.65 ± 2.20^*∗*^	67.87 ± 1.77^*∗*^	19.62 ± 2.90^*∗*^	35.15 ± 3.62^*∗*^	6.56 ± 4.99^*∗*^
3.00	1000	3.67 ± 2.52^*∗*^	9.55 ± 2.48^*∗*^	2.18 ± 0.32^*∗*^	4.15 ± 0.32^*∗*^	−12.66 ± 4.56^*∗*^

**Table 4:** Average IC_50_ of H_2_O_2_ for H9c2(2-1) cells. The IC_50_ value was determined from respective cell viability curves (Figure 6) via GraphPad Prism (GraphPad Software, Inc., USA). Data represent mean ± standard deviation of 3 technical replicates (*n* = 3). “*∗*” denotes significant difference from negative control treated with media + 1% EtOH at *P* ≤ 0.05. Graphical representations of data were depicted in Figure 5.

	Average IC_50 _of H_2_O_2_ (*µ*M)	
	Log [H_2_O_2_]	H_2_O_2_
Control sample		
Negative control (media + 1% EtOH)	2.50 ± 0.01	316.23 ± 1.02
RBE		
BJLN (25 *µ*g/mL)	2.81 ± 0.04^*∗*^	645.65 ± 1.10^*∗*^
BJLN (50 *µ*g/mL)	1.97 ± 0.07^*∗*^	92.90 ± 1.17^*∗*^
MR219 (50 *µ*g/mL)	2.55 ± 0.06^*∗*^	320.63 ± 1.14^*∗*^
MR219 (100 *µ*g/mL)	2.24 ± 0.05^*∗*^	171.79 ± 1.13^*∗*^
