# Corrigendum to “Cytoprotective and Cytotoxic Effects of Rice Bran Extracts in Rat H9c2(2-1) Cardiomyocytes”

**DOI:** 10.1155/2018/3136860

**Published:** 2018-01-22

**Authors:** Xian Wen Tan, Mrinal Bhave, Alan Yean Yip Fong, Eiji Matsuura, Kazuko Kobayashi, Lian Hua Shen, Siaw San Hwang

**Affiliations:** ^1^Faculty of Engineering, Computing and Science, Swinburne University of Technology Sarawak Campus, Sarawak, Malaysia; ^2^Swinburne Sarawak Research Centre for Sustainable Technologies, Swinburne University of Technology Sarawak Campus, Sarawak, Malaysia; ^3^Faculty of Science, Engineering and Technology, Swinburne University of Technology, Melbourne, VIC, Australia; ^4^Department of Cardiology, Sarawak General Hospital, Sarawak, Malaysia; ^5^Clinical Research Centre, Sarawak General Hospital, Sarawak, Malaysia; ^6^Collaborative Research Center (OMIC), Okayama University Graduate School of Medicine, Dentistry, and Pharmaceutical Sciences, Okayama, Japan; ^7^Department of Cell Chemistry, Okayama University Graduate School of Medicine, Dentistry, and Pharmaceutical Sciences, Okayama, Japan

In the article titled “Cytoprotective and Cytotoxic Effects of Rice Bran Extracts in Rat H9c2(2-1) Cardiomyocytes” [1], errors in statistical analyses for inhibitory concentration (IC_50_) have resulted in incorrect tabulations of data for both Tables 2 and 4. The corrected versions of both tables are as below.

Accordingly, in the “Results” (Section 3.1), the text reading “*Based on the results (Table 2), the IC_50_ values of RBE of BJLN were in the range of 61.67 to 64.57 μg/mL over 24, 48, and 72* hours *of incubation time*.” should be corrected to “*Based on the results (Table 2), the IC_50_ values of RBE of BJLN were in the range of 59.57 to 64.27 μg/mL over 24, 48, and 72 hours of incubation time*.”, and “*Based on the results, the IC_50_ values of MR219 RBE were in the range of 95.44 to 111.50 μg/mL over the three different incubation periods (Table 2).*” should be corrected to “*Based on the results, the IC_50_ values of MR219 RBE were in the range of 95.56 to 111.40 μg/mL over the three different incubation periods (Table 2).*”

In addition, in the “Results” (Section 3.3), the text reading “*The positive effects were more distinctive with lower concentrations of RBE (BJLN: 25 μg/mL; MR219: 50 μg/mL) with observable increments in IC_50_ of H_2_O_2_ (BJLN: 645.65 μM; MR219: 320.63 μM) (Table 4) when compared to negative control (316.23 μM). When the two extracts were compared, BJLN (25 μg/mL) extract outran MR219 (50 μg/mL) extract in terms of efficacy with a significant increment in IC_50_ of H_2_O_2_ approximately twofold (645.65 μM)* versus *1.4% (in approximation) when compared to negative control (316.23 μM).*” should be replaced with “*The positive effects were more distinctive with lower concentrations of RBE (BJLN: 25 μg/mL; MR219: 50 μg/mL) with observable increments in IC_50_ of H_2_O_2_ (BJLN: 597.20 μM; MR219: 364.20 μM) (Table 4) when compared to negative control (271.00 μM). When the two extracts were compared, BJLN (25 μg/mL) extract outran MR219 (50 μg/mL) extract in terms of efficacy with a significant increment in IC_50_ of H_2_O_2_ by approximately 2-fold (597.20 μM)* versus *1.4-fold (in approximation) when compared to negative control (271.00 μM).*”, and the text reading “*Significant decrements in the IC_50_ values of H_2_O_2_ were found for cell pretreated with 50 μg/mL BJLN (92.90 μM) and 100 μg/mL MR219 (171.79 μM) extracts when compared to negative control (316.23 μM) (Table 4). The higher concentrations of BJLN and MR219 extracts selected were near the range of IC_50_ of both extracts (IC_50_ of BJLN: 52.18 μg/mL to 73.09 μg/mL; IC_50_ of MR219: 95.44 μg/mL to 111.50 μg/mL).*” should be replaced with “*Significant decrements in the IC_50_ values of H_2_O_2_ were found for cell pretreated with 50 μg/mL BJLN (89.95 μM) and 100 μg/mL MR219 (143.90 μM) extracts when compared to negative control (271.00 μM) (Table 4). The higher concentrations of BJLN and MR219 extracts selected were near the range of IC_50_ of both extracts (IC_50_ of BJLN: 59.57 μg/mL to 64.27 μg/mL; IC_50_ of MR219: 95.56 μg/mL to 111.40 μg/mL)*.”

An incorrect version of Figure 3 with missing graphical elements was published. The corrected version of Figure 3 with the inclusion of graphical elements is as shown below.

Accordingly, Figure 5(b) presented in the original manuscript was also the incorrect version. The fourth datum point for MR219 (50 *μ*g/mL) (grey dotted line) was incorrectly plotted. The correct version of the figure is as shown below with the corrected fourth datum point for MR219 (50 *μ*g/mL) (grey dotted line).

## Figures and Tables

**Figure 3:**
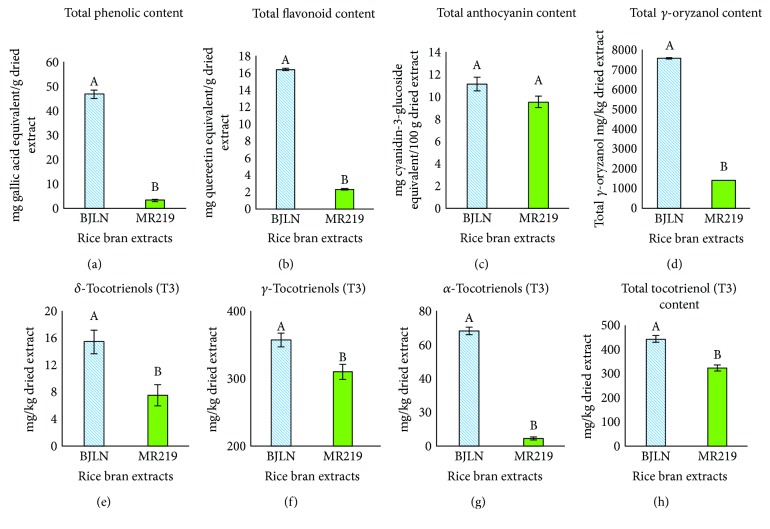
Total contents of selected bioactive compounds in the RBE. Different letters on a bar represent significant differences at *P* ≤ 0.05 (Tukey's test).

**Figure 5:**
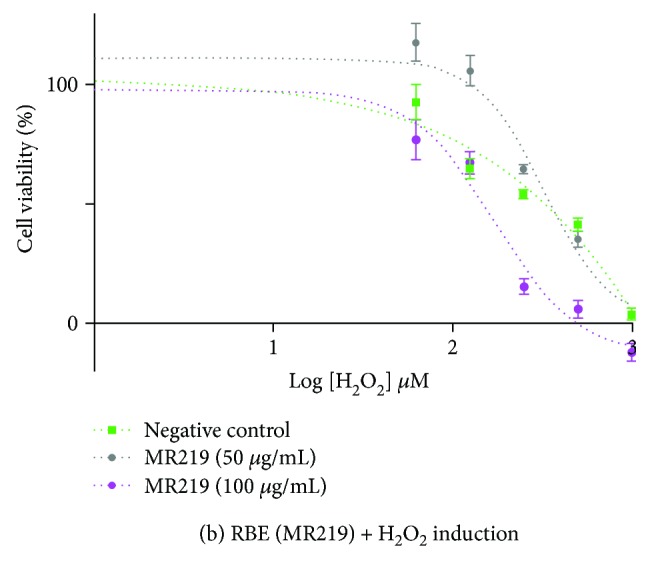
Effects of H_2_O_2_ inductions on cell viabilities of H9c2(2-1) cardiomyocytes pretreated with different concentrations of (a) BJLN RBE (25 *μ*g/mL and 50 *μ*g/mL) and (b) MR219 RBE (50 *μ*g/mL and 100 *μ*g/mL).

**Table 2:** The relative inhibitory concentration (IC_50_) of RBE of BJLN and MR219. Data presented as mean ± standard deviation of three technical replicates (*n* = 3).

	BJLN		MR219	
	Log (dose) (*μ*g/mL)	Dose (*μ*g/mL)	Log (dose) (*μ*g/mL)	Dose (*μ*g/mL)
Day 1 (24 hours)	1.808 ± 0.011	64.27	1.980 ± 0.013	95.56
Day 2 (48 hours)	1.775 ± 0.002	59.57	2.047 ± 0.026	111.40
Day 3 (72 hours)	1.800 ± 0.004	63.10	2.033 ± 0.029	108.00

**Table 4:** Average IC_50_ of H_2_O_2_ for H9c2(2-1) cells. The IC_50_ value was determined from respective cell viability curves ( Figure 5) via GraphPad Prism (GraphPad Software Inc., USA). Data represent mean ± standard deviation of 3 (*n* = 3). ∗ denotes significantly different from negative control treated with media + 1% EtOH at *P* ≤ 0.05. Graphical representations of data were depicted in Figure 5.

	Average IC_50_ of H_2_O_2_ (*μ*M)	
	Log [H_2_O_2_]	H_2_O_2_
Control sample		
Negative control (media + 1% EtOH)	2.433 ± 0.040	271.00
RBE		
BJLN (25 *μ*g/mL)	2.776 ± 0.028^∗^	597.20
BJLN (50 *μ*g/mL)	1.954 ± 0.033^∗^	89.95
MR219 (50 *μ*g/mL)	2.561 ± 0.035^∗^	364.20
MR219 (100 *μ*g/mL)	2.158 ± 0.032^∗^	143.90
