# Supplementary material for: Cytoprotective and Cytotoxic Effects of Rice Bran Extracts in Rat H9c2(2-1) Cardiomyocytes
Source: Oxid Med Cell Longev. 2016 Apr 27;2016:6943053. doi: 10.1155/2016/6943053 (PMC4863109; doi:10.1155/2016/6943053)
Supplement: Supplementary file 1 — Table A represents the data for cell viability of H9c2(2-1) after inductions with different concentrations of hydrogen peroxide (H2O2). Data presented were the mean ± standard deviation of three replicates (n=3). ‘∗' on each column denotes significant differences at P≤0.05 as compared to negative control. Graphical representation of data is illustrated in Fig. 4. [file 6943053.f1.pdf]

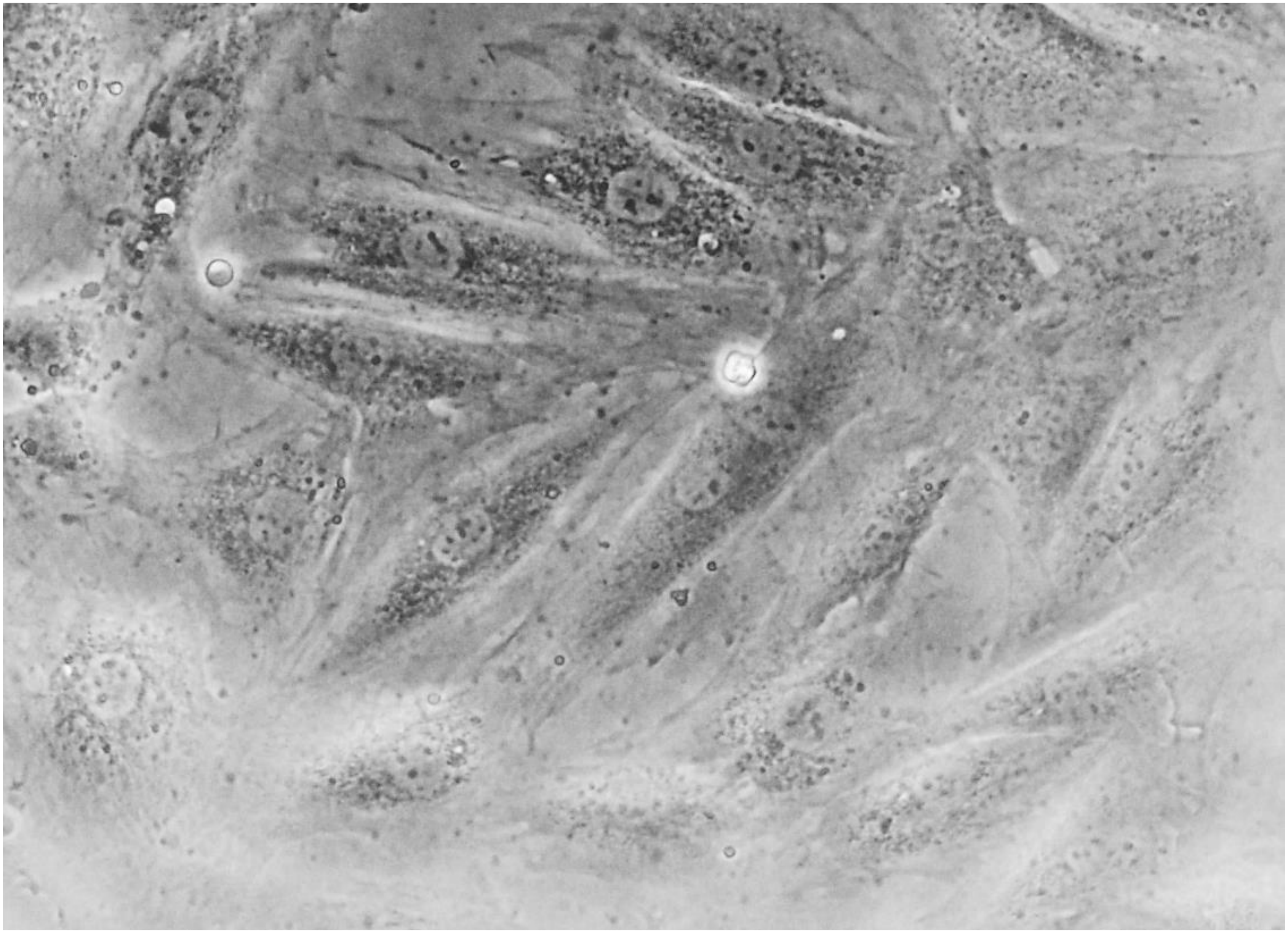

Fig. A: Healthy rat H9c2(2-1) cardiomyocytes. Imaging with an inverted light microscope (Nikon Eclipse T*i*-S: Mag 200x)

Table A: Cell viability of H9c2(2-1) after inductions with different concentrations of hydrogen peroxide (H_2O_2). Data presented were the mean $\pm$ standard deviation of three replicates (n=3). '*' on each column denotes significant differences at $P \leq 0.05$ as compared to negative control. Graphical representation of data is illustrated in Fig. 4.

	H_2O_2 Induction on H9c2(2-1) Cardiomyocytes	
**Log (Dose), µM**	**Dose (µM)**	**Cell Viability (%)**
**1.19**	**15.63**	119.47 ± 3.39*
**1.49**	**31.25**	115.27 ± 5.48*
**1.80**	**62.50**	109.29 ± 2.07*
**2.10**	**125.00**	101.29 ± 3.57
**2.40**	**250.00**	87.56 ± 2.49*
**2.70**	**500.00**	47.68 ± 3.34*
**3.00**	**1000.00**	3.43 ± 1.00*
